# The Effects of Sesame Consumption on Glycemic Control in Adults: A Systematic Review and Meta-Analysis of Randomized Clinical Trial

**DOI:** 10.1155/2021/2873534

**Published:** 2021-10-18

**Authors:** Alireza Yargholi, Mohammad Hasan Najafi, Mohammad Ali Zareian, Jessie Hawkins, Laila Shirbeigi, Mohammad Hossein Ayati

**Affiliations:** ^1^Department of Traditional Medicine, School of Persian Medicine, Tehran University of Medical Sciences (TUMS), Tehran, Iran; ^2^Department of Traditional Medicine, Faculty of Iranian Traditional Medicine, Shahid Sadoughi University of Medical Sciences, Ardakan, Yazd, Iran; ^3^Integrative Health, Franklin School of Integrative Health Sciences, Franklin, Tennessee, USA

## Abstract

**Objectives:**

In recent years, diabetes has become a global health problem that creates a tremendous economic burden for many countries. Clinical trials evaluating the hypoglycemic effects of sesame consumption have produced conflicting results. This systematic review and meta-analysis was conducted to evaluate the effectiveness of sesame as a popular natural herb on glycemic indices in adults.

**Methods:**

The search for related articles in PubMed, Scopus, Google Scholar, and Cochrane library was conducted through May 2021. Results were reported as weighted mean differences (WMD) with 95% confidence intervals (CI) using a random-effects model.

**Results:**

A total of 605 studies were identified through online searching, and a total of eight RCTs representing 382 participants were included in this study. The meta-analyses revealed that sesame consumption significantly decreases serum fasting blood sugar (FBS): (WMD: −28.23 mg/dl; 95% CI (−39.16, −17.13), *I*^2^ = 97.6%; 95% CI (96, 98)), and hemoglobin A1c (HbA1c): (WMD: −1.00%; 95% CI (−1.11, −0.88), *I*^2^ = 0%; 95% CI (0, 79)) as compared to the control group.

**Conclusion:**

This study provides evidence of the hypoglycemic effects of sesame consumption, particularly in diabetic patients. Additional RCTs on sesame and its preparations should be conducted in different populations to increase generalizability.

## 1. Introduction

Diabetes mellitus is one of the most common metabolic disorders, affecting over 420 million individuals worldwide [1]. This condition was responsible for 28.8 million deaths in 2016 [2]. Type 2 diabetes (T2D) is characterized by insulin resistance and is diagnosed by biochemical parameters such as high concentrations of serum blood sugar, insulin, and glycosylated hemoglobin (HbA1C) [3, 4]. Finding a practical approach to reduce the prevalence of diabetes is essential for global public and economic health. Herbal medicine, a component of complementary medicine (CAM), is commonly used to prevent and treat chronic conditions because of its lower costs and reduced side effects [5].

Sesame (*Sesamum indicum* L.) has been used as a traditional plant-based therapy worldwide, most commonly in Asian regions [6]. Sesame seed and its products (oil, flour, and dietary supplement) are good sources of lignan compounds (sesamin, sesamolin, sesamol, and episesamin) [7]. These lignans are responsible for most medicinal actions of sesame, including antioxidant activity [8], anti-inflammatory actions [9], and hypoglycemic [10] effects. Additionally, sesame contains high amounts of other beneficial components such as *α*-tocopherol, polyunsaturated fatty acids (PUFAs), monounsaturated fatty acids (MUFAs), and fiber [11].

The hypoglycemic effects of sesame may be due to the action of its bioactive lignans which have been shown to improve insulin secretion from B cells of the pancreas [12]. Some animal studies have found that sesame consumption could reduce insulin resistance and enhance the glucose metabolism [12, 13]. In human studies, sesame consumption has produced beneficial effects on serum glucose, HbA1C, and insulin concentrations in diabetic patients [14–16]. However, to the best of our knowledge, there has not been a systematic review and meta-analysis examining the effects of sesame consumption on glycemic control. The purpose of this study was to systematically assess all randomized clinical trials (RCTs) performed on the impact of sesame and sesame-based products on glycemic control in adults and conduct a meta-analysis to summarize our findings.

## 2. Materials and Methods

### 2.1. Search Strategy

We used the Preferred Reporting Items for Systematic Reviews and Meta-Analyses (PRISMA) guidelines for this study [17] (Supplementary Table 1).

PubMed/Medline, Cochrane Library, Google Scholar, and Scopus were searched to locate relevant RCTs published through May 2021. The search strategy was prepared using medical subject headings (MeSH) and dependent keywords as follows: ((“Sesame” OR “Sesame Oil” OR “Sesame Seed” OR “Sesamum”) AND (“Blood Glucose” OR “Insulin” OR “Glycated Hemoglobin A” OR “Insulin Resistance” OR “Diabetes Mellitus” OR “Glucose” OR “Blood Glucose” OR “Insulin Resistance” OR “diabetes” OR T2DM OR “hyperglycemia” OR “hyperglycemia” OR “HbA1c” OR “insulin sensitivity” OR “HOMA” OR “glucose homeostasis “OR “insulin secretion” OR “beta-cell function” OR “glycemic control” OR “glucose tolerance” OR “glucose metabolism”)). No restrictions were used when searching the databases. To ensure all related articles were identified, we checked the reference list of all qualified studies and related reviews.

### 2.2. Eligibility Criteria

Two investigators independently browsed the title and abstract of every article and included them if they met the inclusion criteria. To be included, the studies had to be RCTs (parallel or crossover) examining the effect of sesame seed and its preparations (oil/flour/supplement) on glycemic indices such as serum fasting blood sugar (FBS), hemoglobin A1C (HbA1C), insulin concentrations, insulin resistance (HOMA-IR: homeostasis model assessment of insulin resistance), and insulin sensitivity (QUICKI: quantitative insulin sensitivity check index).

Studies that used other compounds in addition to sesame only in the intervention group, studies without a control group, and studies with the same populations were excluded.

### 2.3. Data Extraction

Two researchers independently extracted the following data from every eligible article: author's name, publication year, country, sesame source and quantity, duration of intervention, participant's information (health condition, age, gender, and sample size), study type, and glycemic indices values (mean and standard deviation) before and after the intervention. Discrepancies were solved by consulting with the principal investigator.

### 2.4. Risk of Bias Assessment

Risk of bias was evaluated by two researchers independently using the Jadad checklist [18]. This scale contains three sections: randomization, blinding, and description of dropouts. Studies with score of ≥3 were considered to be high quality.

### 2.5. Quality of Evidence

Quality of the evidence was evaluated using the Grades of Recommendation, Assessment, Development, and Evaluation (GRADE) tool [19]. This tool evaluates within-study risk of bias, inconsistency of results, indirectness of evidence, imprecision, and risk of publication bias. Studies were classified as high, moderate, low, or very low quality.

### 2.6. Statistical Method

Effect sizes were reported as weighted mean differences (WMD) and 95% confidence intervals (CI) and were obtained by combining effect sizes of mean (SD) changes of glycemic control biomarkers using random-effects models. Due to the small number of the studies in these analyses, the Hartung–Knapp adjustment was also used [20]. We calculated mean change and standard deviation using following formula in studies where those values were not reported: mean change = final values−baseline values; SD = square root ((SD baseline) 2 + (SD final) 2–(2R × SD baseline × SD final)) [21]. A correlation coefficient equal to 0.8 was considered the *R* value when calculating SD change [21]. Between study heterogeneity was assessed using the *I*^2^ index and its confidence interval [22]. Low, moderate, and high heterogeneities were ascribed to *I*^2^ values of 25–50%, 50–75%, and >75%, respectively [23]. Predetermined subgroup analyses were conducted on factors including type of sesame product (sesame seed, sesame oil, and sesamin supplement), sample size, duration of intervention, and quality of studies. Sensitivity analyses were performed to determine the influence of each individual study on the overall effect size. Potential publication bias was evaluated using Egger's weighted regression test, as well as visual inspection of funnel plots. All statistical procedures were performed using STATA (Version 12.0, Stata Corp., College Station, TX). Statistical significance was defined as *P* < 0.05.

## 3. Results

### 3.1. Search Results

**Figure 1:**
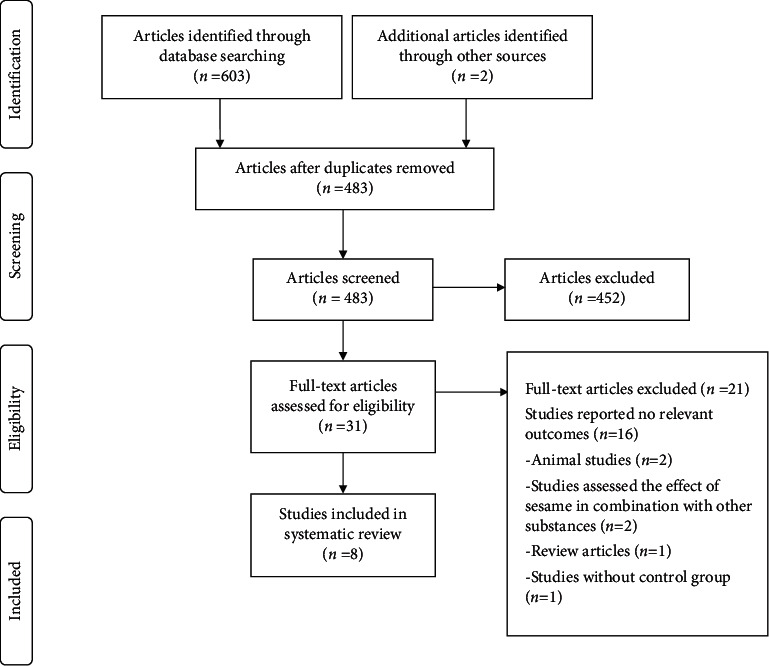
Flow diagram of the included and excluded studies.

A total of 605 articles were entered into Endnote for screening, as shown in Figure 1. After reviewing the titles, abstracts, and subsequently full-texts of remained articles and removing duplicates, eight RCTs [14–16, 24–28] were included in this systematic review.

General characteristics of the included studies are reported in Table 1. These studies were published between 2006 and 2020 and were conducted in Iran [14, 25, 26, 28], India [16, 27], Brazil [24], and Pakistan [15]. The age of the participants and the duration of intervention were 18–70 years and 6–12 weeks, respectively. A total of 262 participants took part in sesame intervention, with 255 in control groups. Only one article was conducted on women [24], and all other trials included males and females [14–16, 25–28]. Sesame was evaluated as sesame oil [15, 16, 26–28], sesame seed [24, 25], and sesamin supplement [14]. Six RCTs had a parallel design [14–16, 24, 25, 28], while two studies had a crossover design [26, 27]. Based on Jadad scale evaluations, four studies were considered to be high-quality [14, 25, 26, 28] (Table 2).

### 3.2. Meta-Analysis

#### 3.2.1. The Effects of Sesame Intake on FBS

**Figure 2:**
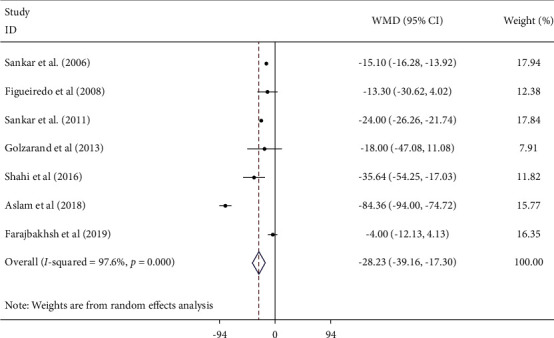
Forest plot showing the effects of sesame consumption on circulating FBS in adults using the random effects model. Values are WMDs (95% CIs) comparing changes in FBS over time between treatment and control groups. CI, confidence interval; FBS, fasting blood sugar; WMD, weighted mean difference.

After pooling seven studies [14–16, 24, 25, 27, 28] evaluating the impact of sesame on FBS by the random effect model, we found that sesame consumption significantly reduces serum FBS levels as compared to a control group (WMD: −28.23 mg/dl; 95% CI (−39.16, −17.13), *I*^2^ = 97.6%; 95% CI (96, 98)) (Figure 2). The results in Hartung–Knapp adjustment analysis are as follows: WMD 28.01 mg/dl; 95% CI (2.31, 53.70), *I*^2^ = 97.5%. When evaluating heterogeneity, sesame preparation, duration of treatment, and sample size were identified as the sources (Table 3).

#### 3.2.2. The Effect of Sesame Intake on HbA1C

**Figure 3:**
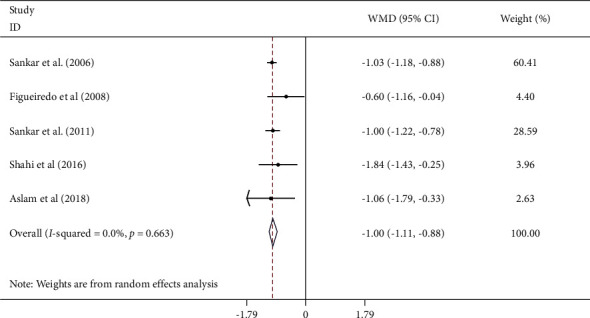
Forest plot showing the effects of sesame consumption on circulating HbA1c in adults using the random effects model. Values are WMDs (95% CIs) comparing changes in HbA1c over time between treatment and control groups. CI, confidence interval; HbA1c, hemoglobin A1c; WMD, weighted mean difference.

Five studies [14–16, 24, 27] that examined the effect of sesame on serum HbA1C levels were used in the meta-analysis. The result of the random effect model was WMD: −1.00%; 95% CI (−1.11, −0.88), *I*^2^ = 0%; 95% CI (0, 79) (Figure 3), and Hartung–Knapp adjustment analysis was WMD 0.99%; 95% CI (0.82, 1.16), *I*^2^ = 0% in the serum HbA_1_C level. Subgroup analysis findings are given in Table 3.

#### 3.2.3. The Effects of Sesame Intake on Other Glycemic Control Markers

Meta-analysis of three studies [14, 15, 28] on the effect of sesame usage on the serum insulin level failed to show any significant results (WMD: random effect model: 7.51 IU/ml; 95% CI (−3.81, 18.83), *I*^2^ = 98.3%%; 95% CI (97, 99), Hartung–Knapp adjustment analysis 7.52 IU/ml; 95% CI (−26.85, 11.81), *I*^2^ = 98.3%).

### 3.3. Sensitivity Analysis

The sensitivity analysis found that none of the studies dramatically influenced the overall findings of FBS, HbA1C, and insulin level (Supplementary Figures 1–3).

### 3.4. Publication Bias

After evaluating Egger test findings and funnel plots, no evidence of publication bias was detected regarding FBS (*P*=0.35), HbA1c (*P*=0.22), and insulin (*P*=0.98) (Supplementary Figures 4–6).

### 3.5. Quality of Evidence

The quality of the evidence was evaluated through GRADE. We found that studies conducted on the effects of sesame supplementation on serum FBS and HbA1c were of low quality and those on insulin were very low quality (Table 4).

## 4. Discussion

This systematic review and meta-analysis study summarizes the findings of eight randomized clinical trials evaluating the effect of sesame consumption on glycemic indices. This study found that sesame consumption has a hypoglycemic effect which decreases serum FBS and HbA1C. The reductions of 28.23 mg/dl in the FBS level and 1.0% of HbA1C are clinically significant. A change (either positive or negative) in the A1C percentage of 0.5% is considered clinically significant [29]. This is important to public health as an increase of 18 mg/dl (1 mmol/l) in glucose levels has been associated with a 4% increase in mortality risk in nondiabetic patients and 5% in diabetic patients [30]. In this analysis, all of the eligible studies were conducted on patients with type two diabetes. Sesame intake has a larger improvement effect on glycemic indices among diabetic patients due to the high serum level of glycemic markers in this group.

We also found that serum FBS and HbA1C declined among people who consumed sesame. This is consistent with findings from an in vitro study on diabetic rats, which produced evidence that sesamin leads to a significant improvement in the serum FBS and HbA1c in a dose-dependent manner by promoting glycogen synthesis in the liver [12]. Similarly, Devarjan et al. in an open-label randomized dietary intervention study investigated the effects of the combination of sesame oil blend (sesame oil along with rice bran oil) (maximum 35–40 ml/d as cooking oil) and glibenclamide (5 mg/d) for 8 weeks on glycemic indices in type 2 diabetes mellitus patients as compared to diabetic patients only used glibenclamide. This study also demonstrated the lowering effects of sesame oil blend and glibenclamide on serum FBS and HbA1C as compared to the glibenclamide group [31]. Furthermore, Mosalaeipour Yazdi et al., in 2008, produced evidence that sesame oil has the ability to improve FBS and HbA1C in type II diabetic patients when they carried out a quasiexperimental study lasting 6 weeks assessing the influence of daily intake of 30 g sesame oil without any control group [32].

These findings differ from a randomized, triple-blind, three-way crossover trial by Raeisi-Dehkordi et al. with 95 subjects who have type 2 diabetes (18–60 years). These patients substituted sesame oil, canola oil, or sesame-canola oil for their regular dietary oil for 9 weeks with a 4-week washout period. When sesame oil or sesame-canola oil was compared to canola oil, no significant effect on FBS, insulin, and HOMA-IR was observed [26]. However, in this study, participants only replaced dietary oils; there was not a set dosage [26]. This variation in consumption levels may explain the inability to find an effect in this trial [26].

These discrepancies might also be explained by the difference in bioavailability among lignan compounds in sesame products (sesame seeds, sesame lignans, and sesame oil). Most of the studies in this analysis used sesame oil. Sesame oil contains greater quantities of lignan (11.51 mg/g) [33] than sesame seed (5.81 mg/g) [34]. Sesame oil also is a rich source of *α*-tocopherol (40 mg/100 g), PUFA (43%), and MUFA (40%) [27]. Epidemiological studies have found that substituting foods high in saturated fatty acids with foods high in MUFA and PUFA could be associated with the prevention of diabetes [35]. High amounts of antioxidant compounds (vitamin E and lignans) in sesame oil may also contribute through powerful radical scavenging effects, protecting B cells from destruction [36].

It is also important to note the lack of consistency regarding placebo substances used for comparison to sesame. These oils (sunflower, canola, soybean, palm, and groundnut oils) contain different levels of PUFA, MUFA, and other biological compounds which can positively influence glycemic markers. It is challenging to quantify the actual effect of sesame on glycemic indices above that of a placebo due to the known therapeutic effect of these oils.

The hypoglycemic effects of sesame may be explained by multiple potential mechanisms. Sesamin can inhibit the augment of blood glucose by playing a role in increasing glycogen production [12]. It has been proposed that sesamin plays a role in glucose uptake, carbohydrate metabolism, and insulin signal transduction pathways by controlling the expression of relevant genes in type 2 diabetic rats [37]. Previous studies have suggested that high levels of dietary MUFA may control glycemic responses by suppressing *β*-cell destruction and elevating insulin sensitivity [38, 39]. The mechanism by which sesame decrease HbA1c is unknown, but the antioxidant activity of sesamin may explain some of the actions [16]. Sesame may protect B cells from death by scavenging reactive oxygen species (ROS) [8].

This study has several limitations. The small number of trials identified in the literature suggests that the present systematic review and meta-analysis results may be biased by the sample size. Similarly, eligible studies were found to be heterogeneous regarding sesame preparation, placebo content, duration of intervention, and sample size.

The overall result may have been influenced by the differences in dosing and sourcing of sesame. Subgroup analysis to identify dose-dependence or contrast groups by dosing was not feasible due to variations in the sesame preparations and the difficulty of obtaining accurate dose conversions.

Additionally, several of the studies in this analysis was found to be of poor quality, limiting the generalizability of these findings. Several trials failed to sufficiently describe the process of randomization, allocation concealment, and blinding. There is also a geographic limitation with most of the eligible studies having been conducted in Asian countries. Finally, the majority of trials did not control for the dietary changes of participants during the intervention, which may have influenced the outcome.

The protocol of this study has not been registered in PROSPERO. However, PROSPERO and all relevant databases were comprehensively searched to confirm that no systematic review and meta-analysis has yet been conducted.

## 5. Conclusion

This systematic review produces evidence that sesame consumption could decrease glycemic indices, especially in diabetic patients. This study finds that sesame is a safe and effective complementary approach to improve hyperglycemia in clinical practice. However, the small numbers of related RCTs and high amounts of heterogeneity among these studies limit generalizability. We recommend that additional high-quality RCTs be conducted on participants with different health conditions, varying duration of intervention, and multiple kinds and dosages of sesame consumption to confirm our findings.

## Figures and Tables

**Table 1:** Characteristics of randomized trials on the effects of sesame consumption on glycemic indices in adults included in the meta-analysis^3^.

Reference	Publication year	Location	Subjects and gender	Age, *y*^1^	Design	Intervention type		Duration (wk)	Notes about subjects	Outcome	Outcome^2^		Findings
						Intervention	Control				Intervention	Control	
Farajbakhsh et al. [28]	2019	Iran	M: 16 F: 31 S: 24 (7/17) C: 23 (9/14)	30–70 y S: 48.04 ± 7.67 C: 50.17 ± 7.6	Parallel	Sesame oil (30 ml/d)	Placebo (sunflower oil)	8	Patients with metabolic syndrome	FBS Insulin HOMA-IR	FBS (mg/dl): Before: 112.58 ± 29.52 After: 108.3 ± 28.0 Insulin (mU/l): Before: 8.25 ± 4.21 After: 7.7 ± 3.6 HOMA-IR: Before: 2.33 ± 1.65 After: 2.1 ± 1.4	FBS (mg/dl): Before: 106.9 ± 14.5 After: 106.7 ± 12.8 Insulin (mU/l): Before: 8.3 ± 6.0 After: 7.3 ± 5.0 HOMA-IR: Before: 2.14 ± 1.4 After: 2.11 ± 1.2	Sesame consumption significantly decreased FBS compared to the control group

Mohammad Shahi et al. [14]	2016	Iran	F + M: 48 S: 24 C: 24	30–60 y S: 50 ± 12.3 C: 51.72 ± 12.24	Parallel	Sesamin capsule (200 mg/d)	Placebo capsules (starch)	8	Type 2 diabetes mellitus	FBS HbA1C Insulin HOMA-IR	FBS (mg/dl): Before: 172.5 ± 53.09 After: 138.59 ± 36.89 HbA1C (%): Before: 8.28 ± 1.55 After: 7.51 ± 1.14 Insulin (mU/l): Before: 32.99 ± 9.71 After: 36.78 ± 18.57 HOMA-IR: Before: 13.24 ± 6.11 After: 12.73 ± 6.32	FBS (mg/dl): Before: 145.4 ± 49.53 After: 147.13 ± 54.97 HbA1C (%): Before: 7.76 ± 1.77 After: 7.83 ± 1.85 Insulin (mU/l): Before: 40.56 ± 25.35 After: 37.28 ± 18.61 HOMA-IR: Before: 12.72 ± 6.97 After: 12.19 ± 7.06	Sesame consumption significantly decreased FBS and HbA1C compared to the control group
Aslam et al. [15]	2018	Pakistan	F + M: 46 S: 23 C: 23	18–60 y	Parallel	Sesame oil (30 ml/d)	Placebo (soybean oil)	12	Type 2 diabetes mellitus	FBS HbA1C Insulin	FBS (mg/dl): Before: 189.09 ± 4.42 After: 137.83 ± 3.16 HbA1C (%): Before: 7.55 ± 0.37 After: 6.96 ± 0.26 Insulin (mU/l): Before: 12.26 ± 1.24 After: 23.13 ± 1.15	FBS (mg/dl): Before: 185.04 ± 6.84 After: 218.14 ± 5.92 HbA1C (%): Before: 7.55 ± 0.37 After: 8.02 ± 0.37 Insulin (mU/l): Before: 11.97 ± 0.81 After: 7.93 ± 0.38	Sesame consumption significantly decreased FBS and HbA1C and increased insulin compared to the control group

Sankar et al. [16]	2011	India	F + M: 42 S: 20 C: 22	S: 58 ± 3 C: 58 ± 4	Parallel	Sesame oil (35 g/d) + glibenclamide (5 mg/d)	Glibenclamide (5 mg/d)	8	Type 2 diabetes mellitus	FBS HbA1C	FBS (mg/dl): Before: 150 ± 5.6 After: 96 ± 4.6 HbA1C (%): Before: 5.2 ± 0.6 After: 3.02 ± 0.2	FBS (mg/dl): Before: 150 ± 6.7 After: 120 ± 5.6 HbA1C (%): Before: 5.4 ± 0.4 After: 4.22 ± 0.4	Sesame plus glibenclamide consumption significantly decreased FBS and HbA1C compared to the glibenclamide group

Sankar et al. [27]	2006	India	F + M: 40 S: 40 C: 40	45–65 y S: 56.63 ± 7.3 C: 56.63 ± 7.3	Crossover	Sesame oil (35 g/d)	Placebo (palm or groundnut oils)	6	Type 2 diabetes mellitus + hypertension	FBS HbA1C	FBS (mg/dl): Before: 146.4 ± 4.04 After: 127.4 ± 4.2 HbA1C (%): Before: 5.97 ± 0.5 After: 4.15 ± 0.2	FBS (mg/dl): Before: 146.4 ± 4.04 After: 142.5 ± 4.6 HbA1C (%): Before: 5.97 ± 0.5 After: 5.18 ± 0.26	Sesame consumption significantly decreased FBS and HbA1C compared to the control group

Figueiredo et al. [24]	2008	Brazil	F: 28 S: 14 C: 14	30–65 y	Parallel	Sesame flour (30 g/d)	Control	8	Type 2 diabetes mellitus	FBS HbA1C	FBS (mg/dl): Before: 148.2 ± 45.2 After: 127 ± 22.3 HbA1C (%): Before: 8.4 ± 1.3 After: 8 ± 0.9	FBS (mg/dl): Before: 129 ± 13.4 After: 121.1 ± 20.8 HbA1C (%): Before: 8 ± 0.1 After: 8.2 ± 0.8	There was no significant difference at FBS and HbA1C levels between sesame and control groups
Raeisi-Dehkordi et al. [26]	2020	Iran	F + M: 95 F: 49 M: 46 S: 92 Ca: 95 S + Ca: 95	18–60 y S: 49.17 ± 0.7 Ca: 49.17 ± 0.7 S + Ca: 49.17 ± 0.7	Crossover	Sesame oil Sesame oil + canola oil	Canola oil Canola oil	9	Type 2 diabetes mellitus	FBS Insulin HOMA-IR-QUICKI	FBS (mg/dl): Before: 116.04 ± 2.42 Change: 1.59 ± 2.11 Insulin (mU/l): Before: 17.01 ± 0.97 Change: −6.0 ± 1.72 HOMA-IR: Before: 2.28 ± 0.11 Change:−0.72 ± 0.20 FBS (mg/dl): Before: 116.85 ± 2.74 Change: −2.53 ± 3.26 Insulin (mU/l): Before: 16.10 ± 0.63 Change: −5.03 ± 1.54 HOMA-IR: Before: 2.16 ± 0.008 Change: −0.62 ± 0.18 QUICKI: Before: 0.3 ± 0.002 Change: 0.009 ± 0.003 QUICKI: Before: 0.31 ± 0.002 Change: 0.009 ± 0.003	FBS (mg/dl): Before: 122.64 ± 3.52 Change: 7.72 ± 3.15 Insulin (mU/l): Before: 17.25 ± 0.74 Change: −2.68 ± 1.36 HOMA-IR: Before: 2.34 ± 0.1 Change: −0.25 ± 0.17 QUICKI: Before: 0.3 ± 0.002 Change:−0.002 ± 0.003	There was no significant difference at FBS, insulin, HOMA-IR, and QUICKI levels between sesame as well as sesame-canola and control groups

Golzarand et al. [25]	2013	Iran	F + M: 36 F:28 M:8 S:20 (16/4) C:16 (12/4)	S: 50 ± 10 C: 53 ± 9	Parallel	Grounded sesame seed (28 g/d)	Control	6	Type 2 diabetes mellitus	FBS	FBS (mg/dl): Before: 148 ± 53 After: 137 ± 9.2	FBS (mg/dl): Before: 151 ± 52 After: 158 ± 12.3	There was no significant difference at the FBS level between sesame and control groups

^1^Values are overall ranges and means ± SDs/SEs in each group. ^2^Values are means ± SDs/SEs. ^3^Ca, canola; C, control; F, female; FBS, fasting blood sugar; HbA1C, glycosylated hemoglobin; HOMA-IR, homeostasis model assessment of insulin resistance; M, male; S, sesame; QUICKI, quantitative insulin sensitivity check index.

**Table 2:** Jadad risk of bias assessment for randomized controlled trials on the effect of sesame consumption on diabetic indices in adults.

Reference	Randomization	Method of randomization	Blinding of participants, personnel, and outcome assessors	Method of blinding	An account of all patients	Total score
Farajbakhsh et al.	^*∗*^		^*∗*^		^*∗*^	3
Mohammad Shahi al.	^*∗*^		^*∗*^	^*∗*^	^*∗*^	4
Aslam et al.	^*∗*^				^*∗*^	2
Sankar et al.	^*∗*^				^*∗*^	2
Sankar et al	^*∗*^				^*∗*^	2
Figueiredo et al	^*∗*^				^*∗*^	2
Raeisi-Dehkordi et al.	^*∗*^	^*∗*^	^*∗*^	^*∗*^	^*∗*^	5
Golzarand et al.	^*∗*^		^*∗*^		^*∗*^	3

**Table 3:** Pooled estimates of the effects of sesame supplementation on glycemic control biomarkers within different subgroups^3^.

	Number of trials		WMD (95% CI)		*P* value		*P* heterogeneity		*I* ^2^ (%)	
	FBS	HbA1c	FBS	HbA1c	FBS	HbA1c	FBS	HbA1c	FBS	HbA1c
Total	7	5	−28.22 (−39.15, −17.29)	−1.00 (−1.11, −0.88)	<0.001	<0.001	<0.001	0.663	97.6	0

Type of intervention										
Sesame capsule	1	1	−35.64 (−54.25, −17.02)	−0.84 (−1.43, −0.24)	<0.001	0.005	—	—	—	—
Sesame seed	2	1	−14.53 (−29.41, 0.35)	−0.60 (−1.16, −0.03)	0.056	0.036	0.786	—	0	—
Sesame oil	4	3	−30.88 (−44.33, −17.42)	−1.02 (−1.14, −0.89)	<0.001	<0.001	<0.001	0.971	98.8	0
Duration of treatment										
<8 wk	2	1	−15.10 (−16.28, −13.92)	−1.03 (−1.18, −0.87)	<0.001	<0.001	0.845	—	0	—
=8 wk	4	3	−18.38 (−31.58, −5.19)	−0.93 (−1.12, −0.74)	0.006	<0.001	<0.001	0.407	87.8	0
>8 wk	1	1	−84.36 (−94.00, −74.72)	−1.06 (−1.78, −0.33)	<0.001	0.004	—	—	—	—
Sample size										
<40	2	2	−14.53 (−29.41, 0.35)	−0.90 (−1.28, −0.52)	0.056	<0.001	0.786	0.147	0	52.3
≥40	5	3	−31.58 (−43.98, −19.19)	−0.98 (−1.18, −0.78)	<0.001	<0.001	<0.001	0.866	98.4	0
Study quality										
High	3	1	−18.12 (−40.39, 4.14)	−0.84 (−1.43, −0.24)	0.111	0.005	0.008	—	79.3	—
Low	4	4	−34.32 (−48.25, −20.40)	−1.00 (−1.12, −0.88)	<0.001	<0.001	<0.001	0.547	98.7	0

CI, confidence interval; FBS, fasting blood sugar; HbA1c, hemoglobin A1c; WMD, weighted mean difference.

**Table 4:** GRADE evidence profile for the effects of sesame supplementation on diabetic indices in adults^1^.

Certainty assessment							MD (95% CI)	Certainty	Importance
Outcome	No. of studies	Risk of bias	Inconsistency	Indirectness	Impression	Other considerations			
FBS, mg/dl	7	Serious	Nonserious	Serious	Nonserious	Non	−28.22 (−39.15, −17.29)	⊕⊕○○ Low	Important
HbA1c, %	5	Serious	Nonserious	Serious	Nonserious	Non	−1.00 (−1.11, −0.88)	⊕⊕○○ Low	Important
Insulin, IU/ml	3	Nonserious	Serious	Serious	Serious	Non	7.51 (−3.81, 18.83)	⊕○○○ Verylow	Important

^1^CI, confidence interval; GRADE, Grades of Recommendations, Assessment, Development, and Evaluation; FBS, fasting blood sugar; HbA1c, hemoglobin A1c; MD, mean difference.

## Supplementary Materials

Supplementary Materials Supplementary Figure 1: analysis of the influence of sesame consumption on serum FBS concentrations in adults. CI, confidence interval; FBS, fasting blood sugar. Supplementary Figure 2: analysis of the influence of sesame consumption on serum HbA1c concentrations in adults. CI, confidence interval; HbA1c, hemoglobin A1c. Supplementary Figure 3: analysis of the influence of sesame consumption on serum insulin concentrations in adults. CI, confidence interval. Supplementary Figure 4: funnel plot for assessing publication bias in the studies reporting the effects of sesame consumption on serum FBS concentrations in adults. FBS, fasting blood sugar; SE, standard error; WMD, weighted mean difference. Supplementary Figure 5: funnel plot for assessing publication bias in the studies reporting the effects of sesame consumption on serum HbA1c concentrations in adults. HbA1c, hemoglobin A1c; SE, standard error; WMD, weighted mean difference. Supplementary Figure 6: funnel plot for assessing publication bias in the studies reporting the effects of sesame consumption on serum insulin concentrations in adults. SE, standard error; WMD, weighted mean difference.
