# Supplementary material for: The Effects of Sesame Consumption on Glycemic Control in Adults: A Systematic Review and Meta-Analysis of Randomized Clinical Trial
Source: Evid Based Complement Alternat Med. 2021 Oct 18;2021:2873534. doi: 10.1155/2021/2873534 (PMC8545509; doi:10.1155/2021/2873534)
Supplement: Supplementary Materials — Supplementary Figure 1: analysis of the influence of sesame consumption on serum FBS concentrations in adults. CI, confidence interval; FBS, fasting blood sugar. Supplementary Figure 2: analysis of the influence of sesame consumption on serum HbA1c concentrations in adults. CI, confidence interval; HbA1c, hemoglobin A1c. Supplementary Figure 3: analysis of the influence of sesame consumption on serum insulin concentrations in adults. CI, confidence interval. Supplementary Figure 4: funnel plot for assessing publication bias in the studies reporting the effects of sesame consumption on serum FBS concentrations in adults. FBS, fasting blood sugar; SE, standard error; WMD, weighted mean difference. Supplementary Figure 5: funnel plot for assessing publication bias in the studies reporting the effects of sesame consumption on serum HbA1c concentrations in adults. HbA1c, hemoglobin A1c; SE, standard error; WMD, weighted mean difference. Supplementary Figure 6: funnel plot for assessing publication bias in the studies reporting the effects of sesame consumption on serum insulin concentrations in adults. SE, standard error; WMD, weighted mean difference. [file 2873534.f1.docx]

**Supplementary Figure 1.** Analysis of the influence of sesame consumption on serum FBS concentrations in adults. CI, confidence interval; FBS, Fasting blood sugar

**Supplementary Figure 2.** Analysis of the influence of sesame consumption on serum HbA1c concentrations in adults. CI, confidence interval; HbA1c, Hemoglobin A1c

**Supplementary Figure 3.** Analysis of the influence of sesame consumption on serum insulin concentrations in adults. CI, confidence interval

**
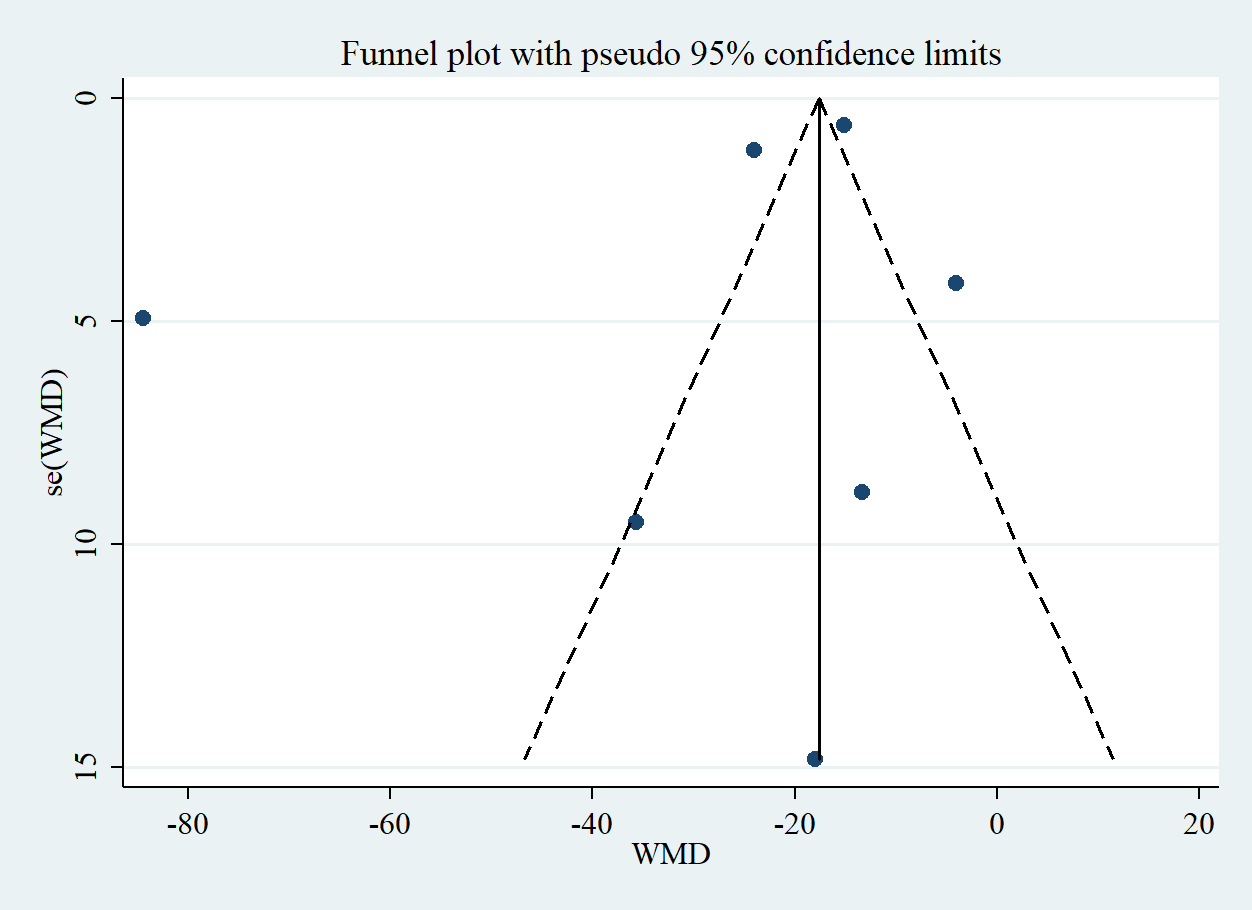
**

**Supplementary Figure 4.** Funnel plot for assessing publication bias in the studies reporting the effects of sesame consumption on serum FBS concentrations in adults. FBS, Fasting blood sugar; SE, standard error; WMD, weighted mean difference

**
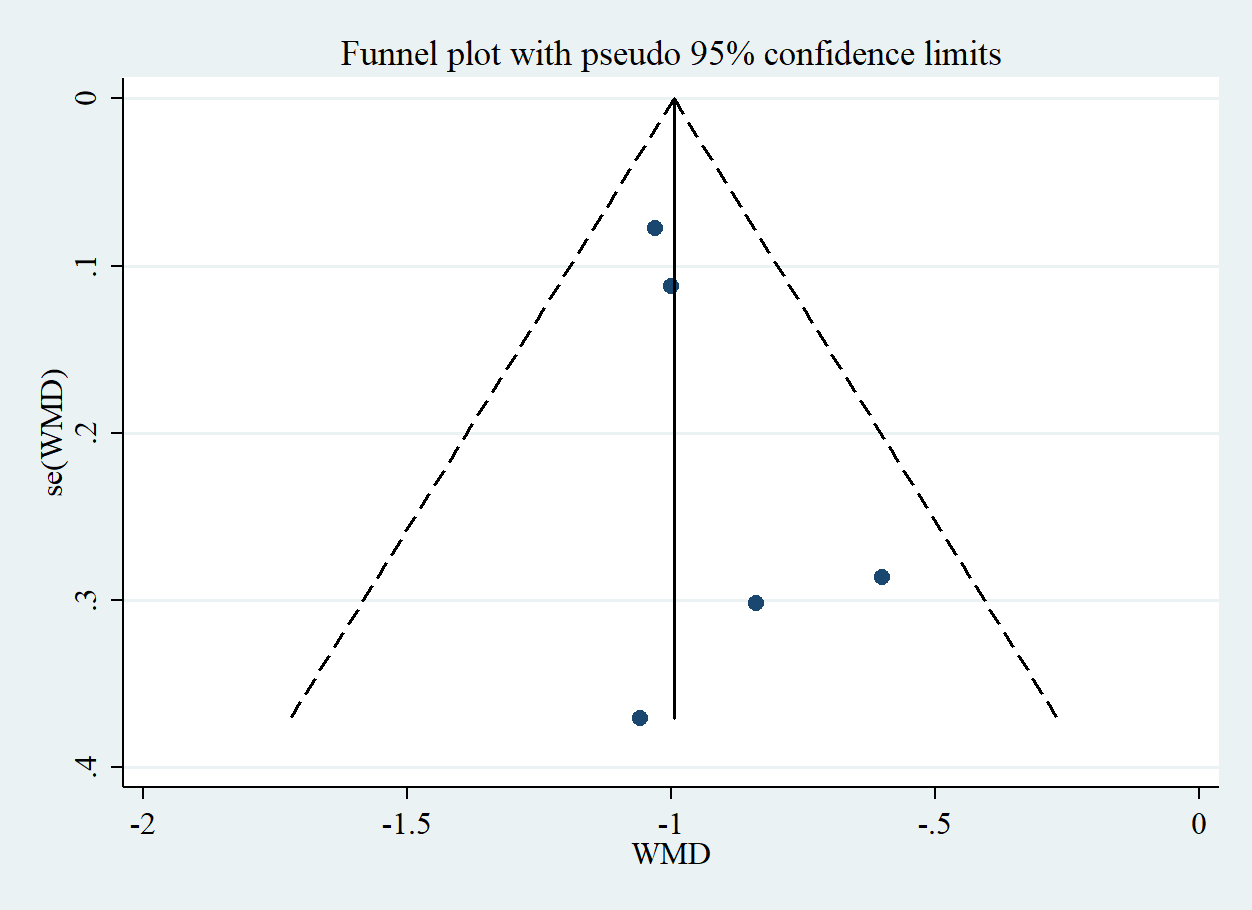
**

**Supplementary Figure 5.** Funnel plot for assessing publication bias in the studies reporting the effects of sesame consumption on serum HbA1c concentrations in adults. HbA1c, Hemoglobin A1c; SE, standard error; WMD, weighted mean difference


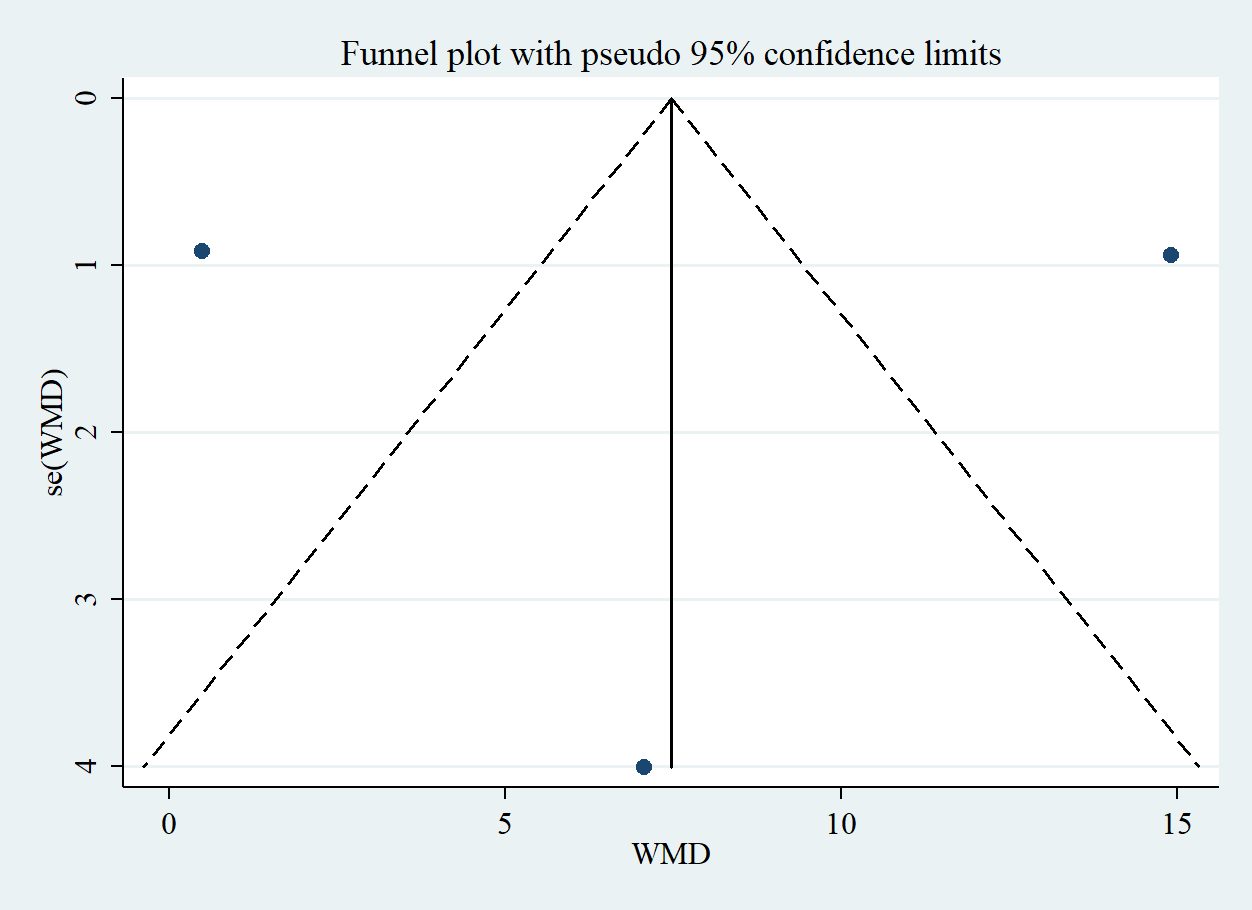


**Supplementary Figure 6.** Funnel plot for assessing publication bias in the studies reporting the effects of sesame consumption on serum insulin concentrations in adults. SE, standard error; WMD, weighted mean difference
